# Bibliometric Analyses of Web of Science Illuminate Research Advances of Neuropterida

**DOI:** 10.3390/insects13050464

**Published:** 2022-05-16

**Authors:** Shuo Tian, Yuxin An, Ruyue Zhang, Liming Wang, Yuyu Wang

**Affiliations:** College of Plant Protection, Hebei Agricultural University, Baoding 071001, China; shuotian139@126.com (S.T.); aaanyuxin@126.com (Y.A.); ruyue_zhang@126.com (R.Z.)

**Keywords:** Neuropterida, *Chrysopa pallens*, Neuroptera, CiteSpace, knowledge graph, bibliometric

## Abstract

**Simple Summary:**

Neuropterida is a relatively primitive group of Holometabola including about 6500 extant species. Many species of this group are natural enemies and can prey on a variety of agricultural pests. This research analyzed the relevant literature in the core database of Web of Science by using CiteSpace, and then summarized the beginning and development of the research in the field of Neuropterida. The results showed that the United States and China had the most publications on the Neuropterida and were the main countries for research. These two countries have had the most productive authors and institutions. Representative institutions for research on natural enemies of Neuropterida included the China Agricultural University and the Chinese Academy of Agricultural Sciences. Due to the late start of Neuropterida research in China, the early research focused on practical applications. In addition, there is more cross-research in the fields of agriculture and biochemistry, molecular biology, chemistry, etc., indicating that the research of Neuropterida can be covered from the macroscopic study to the microscopic study. In addition, it was found that the early research focused on the biological control of Neuropterida by analyzing the keyword burst, whereas the current research has focused on the phylogeny of Neuropterida more.

**Abstract:**

Neuropterida is a relatively primitive group of Holometabola. There are about 6500 extant species. Many species of this group are natural enemies and can prey on a variety of agricultural pests. In order to understand the leading research institutions, researchers and research contents, and to predict the future research directions of Neuropterida, the Web of Science core database, from January 1995 to September 2021, was searched with the theme of “Neuropterida or Neuroptera or Megaloptera or Raphidioptera or Lacewing”. The results showed that the United States and China published relatively more publications than other countries. In addition, researchers from these two countries had more cooperation with other countries. China Agricultural University ranked the highest in the number of publications and centrality in this field. In addition, it was found that the early research focused on the biological control of Neuropterida by analyzing the keyword burst, whereas the more recent research focused on the phylogeny of Neuropterida. As the first representative chromosome-level genome of Neuropterida has been published, the future research of Neuropterida will focus on the genomic studies and molecular mechanisms of their morphological characters, behavior, historical evolution and so on.

## 1. Introduction

Neuropterida is one of the earliest ancient groups of Holometabola that consists of Neuroptera, Raphidioptera and Megaloptera. There are more than 6500 extant species known all over the world [1]. Although the number of species of extant Neuropterida is relatively small and the distribution pattern is discontinuous, the fossil record is very rich, indicating that the population of Neuropterida has evolved over a long period of time and can be treated as living fossils [2]. The use of the fossil records of Neuropterida can help researchers to understand the difficult problems in their geographic distribution and phylogeny. For example, Engel and Grimaldi studied the characteristics of venation and genitalia in the fossils of Neuropterida in 2007, demonstrated that the fossils were distributed discontinuously all over the world and discussed the patterns of their larvae and adults, which promoted the study of the ecological significance of Neuropterida [3]. In addition, Neuropterida also has a relatively high morphological and biological diversity [2]. There are many studies of classification and phylogeny of Neuropterida published. For instance, there were 75 species in 1979 and 101 species in 2019 in Canada, according to Canadian fauna statistics: thus, an increase of 26 species (35%) [4]. Aspöck et al. conducted a phylogenetic study using a computerized cladistic analysis based on 36 adult and larval morphological characteristics at the ordinal level for the first time to upset the conventional Megaloptera and Raphidioptera hypothesis [2]. Later, Aspöck introduced and discussed the phylogeny of Neuropterida in 2002 comprehensively [5]. It was shown that the phylogenetic analysis of Mantispidae based on morphological characteristics reveals a pattern of raptorial foreleg evolution across the family [6]. Aspöck also discussed the phylogeny through the analysis of the genital structures of Neuropterida in 2008 [7]. Mitochondrial data have been widely used in phylogenetic analyses of Neuropterida [8,9,10,11]. For example, to study the origin and variety of lacewings, Wang et al. (2017) undertook phylogenetic analyses based on mitochondrial genomes of Neuropterida [10]. Anchored hybrid enrichment (AHE) data and transcriptome data have also been used to analyze the phylogeny of Neuropterida [12,13,14]. Winterton et al. (2018) recovered a relatively comprehensive phylogeny of Neuropterida using the anchored hybrid enrichment (AHE) data [12]. Machado et al. (2018) proposed a new classification of the antlions by using AHE data [13]. Wang et al. (2019) as well as Alexandros et al. (2020) reconstructed the phylogeny of Neuropterida using transcriptome data [14,15]. Nevertheless, the classification and phylogeny of Neuropterida have not been clarified clearly up to now.

Neuropterida also plays a very important role in biological control. For example, green lacewings can prey on different kinds of agricultural pests, such as aphids, coccids, thrips, planthoppers and whiteflies [16,17,18]. Myrmeleontidae larvae are commonly known as antlions, which can drag psyllid, aphids, caterpillars and other pests into sand to eat [19]. In addition, the third largest group of Neuroptera, brown lacewings, can prey on aphids, mites, scale insects and other small soft pests [20]. With the development of molecular technology, more and more studies focused on the gene functions and the molecular mechanisms of lacewings serving as excellent natural enemies have been conducted, such as a study on the strong prey ability of *Chrysopa pallens* to powerfully perceive information in the environment [21]. The fertility of *C. pallens* was certified to be closely related to a variety of factors, such as female weight and the activities of trypsin-like enzymes [22]. Wang et al. (2021) sequenced and published the first chromosome-level genome of *C. pallens* as the first representative of Neuropterida, which reveals the potential molecular mechanisms as an excellent biocontrol agent [23].

Up to now, most of the related publications of Neuropterida focused on the research of classification and phylogeny as well as biological control [16,24,25,26,27,28,29]. However, there are few reports on the development, history and overview of the research field of Neuropterida. Facing a large number of publications, there are some limitations in the classification and summary by traditional reading methods. In recent years, with the improvement of computation and bibliometrics, the use of software for visual processing and analysis of publications has become more and more popular. The scientific knowledge map shows the development structure and progress of scientific knowledge using visualization technology through a series of methods, such as data mining, information analysis and graph drawing. It can provide practical and valuable references for subject research [30].

In order to provide a theoretical basis for the future research related to Neuropterida, the bibliometric method was used to clarify the basic background, research status and research trends of Neuropterida by analyzing the related publications from January 1995 to September 2021.

## 2. Materials and Methods

### 2.1. Data Collection

The data used in this study were from the Web of Science (WoS). “Neuropterida or Neuroptera or Megaloptera or Raphidioptera or Lacewing” were used as the subject terms for data retrieval. The timespan for the search was from January 1995 to September 2021. Firstly, 2792 publications were identified. Then, 2626 publications were obtained and 166 publications were deleted after removing duplicates. Finally, they were imported into CiteSpace 5.8R2 for analysis.

### 2.2. Analysis

CiteSpace was used to analyze the literature data, which fosters to show the research progress of Neuropterida knowledge domains [31,32]. It is a practical software to methodically learn about one field quickly using the method of co-citation clusters and then to form pivotal points using time-sliced snapshots. Frequency refers to the direct statistics based on the occurrence frequency or citation frequency of nodes. The calculation of intermediate centrality is as follows:

$$\mathrm{Centrality}\;({\mathrm{node}}_{i})=\sum_{s\neq j\neq t}\frac{n_{st}^{i}}{g_{st}}$$

In the Equation, $g_{st}\;$ is the number of the shortest paths from node s to node t and $n_{st}^{i}$ is the number of the shortest paths from node s to node t through node i. From the perspective of information transmission, the higher the centrality of the intermediary, the greater the importance of nodes, which would cause the greatest impact on the network transmission to remove these points.

Firstly, basic parameters were set properly before methodically analyzing the tendency and research status in order to exactly describe the co-occurrence, relationship and citation information. The time slicing in CiteSpace was set as one year per slice, and the top 50 levels of the most cited and high-frequency items from each slice were extracted. Then, the following specific functions were chosen to assess the related review results: (1) subject categories, (2) literature co-citation network, (3) author co-citation, (4) journal co-citation, (5) country collaboration, (6) institution collaboration, (7) author collaboration, (8) keywords analysis and (9) emerging trends.

## 3. Results

### 3.1. Publication Years

**Figure 1:**
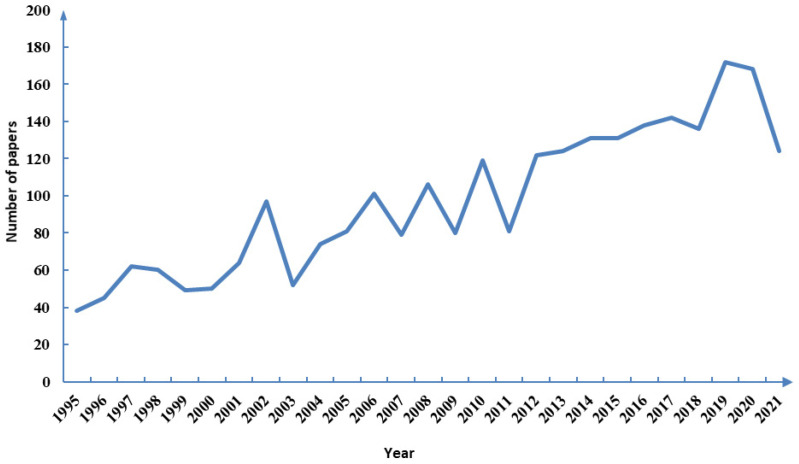
Annual trend chart of publications.

By analyzing the number of publications through the years, we could understand the research history of Neuropterida to a certain extent. Although there are both increases and decreases of the annual number of related publications from January 1995 to September 2021, they generally showed a trend of substantial growth (Figure 1). There were four times when there were distinct large increases in succession (2002, 2010, 2012 and 2019), indicating that the research of Neuropterida was relatively hot and had rapid development in the past twenty years. Among them, the number of publications in 2012 increased the most, with an increase of 41 publications compared to the previous year; however, it did not continue. The reason may be the popularity of high-throughput sequencing technology, which is used by many researchers to solve some former difficult problems. The number of publications in 2003, 2007, 2009, 2011 and 2018 decreased compared to the previous year, indicating the research entered a stable phase. In general, Neuropterida became a research hotspot and the number of publications continued to increase steadily after 2000. The number of publications in 2019 was the highest with a total of 172 publications. The reason may be that Engel et al. summarized and discussed the existing problems of phylogeny and evolution of Neuropterida in 2018, influencing the future research direction of Neuropterida [33]. For example, they suggested that we should pay attention to the in-depth study of Neuropterida using internal anatomy and fossil data. Thus, researchers have a direction for the study of Neuropterida.

### 3.2. Category Network Visualization

**Figure 2:**
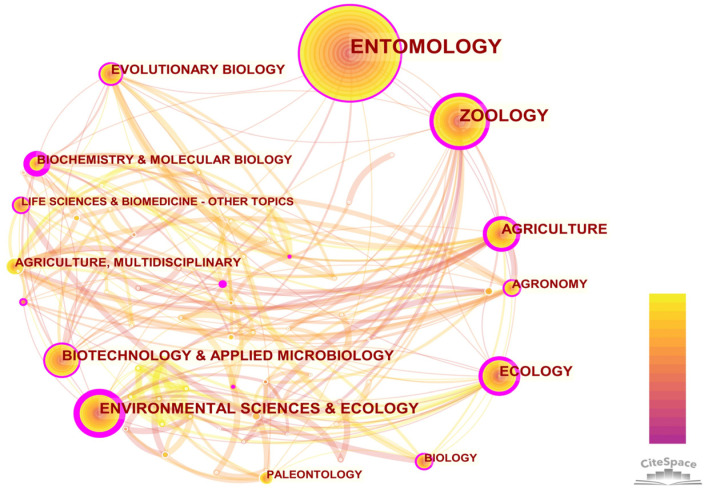
Subject categories co-occurrence network. The node size represents the subject categories’ publication frequency, and the connection line between nodes represents co-occurrence. High-centrality nodes are marked by purple circles.

The categories were visualized to show the relationships of different subjects in this domain. Each publication was distributed to one or more subject categories. This information can be used to analyze which subjects are covered by the publications about Neuropterida. The research in the field of Neuropterida was mainly intersected with entomology and ecology (Figure 2). The thicker the line, the deeper the relationship between the two categories, and the darker the line, the earlier the relationship between the two categories was established. In addition, there was more cross-research in the fields of agriculture and biochemistry, molecular biology, chemistry and so on. It indicated that the research of Neuropterida could be covered from the macroscopic study to the microscopic study. The intersection of multidisciplinary fields put forward higher requirements for the reservation of knowledge of current researchers. This is because, before the popularization of sequencing technology, we could only study the agricultural and ecological significance of Neuropterida based on the morphological characteristics. Now, we can explain its ecological significance from the molecular level. For example, the reproduction of *C. pallens* was proven to be closely related to the activities of trypsin-like and chymotrypsin-like enzymes [22]. This requires us to master not only the morphological data of Neuropterida but also a series of molecular data. Environmental sciences and ecology had a lot of collaboration on Neuropterida and it lasted a long time. Among them, environmental science, agriculture and ecology had relatively high centrality. It indicated that Neuropterida had important research values in these disciplines and the degree of correlation was also high.

### 3.3. The Co-Citation Network Based on the Focus Topic

#### 3.3.1. Literature Co-Citation Analysis

**Figure 3:**
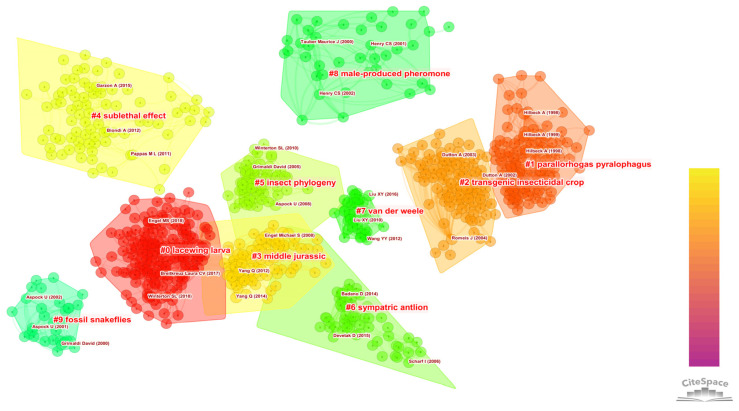
Cluster visualization based on a document co-citation network. The red font represents the clustering label. Different clusters have different regions.

The literature co-citation network was created (Figure 3). 178 unique nodes, 4907 links and 10 main clusters were created with the Q of 0.8056 and the S of 0.8629. The top 10 cited references on Neuropterida are presented in Table 1. A node with high centrality means that it plays an important role in connecting other nodes. Nine articles were related to the phylogeny of Neuropterida and one article was related to the ecological significance of Neuropterida. In order of centrality, the first publication was published by Romeis et al. (2004), which drew a conclusion that the risk posed by predators preying on transgenic corn with high levels of toxins is negligible [34]. The second was by Dutton et al. (2002), which accessed the ecological effects of transgenic corn on the larvae of *Chrysoperla carnea* by the study of three factors: (1) the performances of transgenic corn and non-transgenic corn eaten by three kinds of pests; (2) the intake of the toxin by the three kinds of insects; and (3) the effects on *C. carnea* by feeding them the three different kinds of insects [35]. Other studies have focused on the phylogeny of Neuropterida through diverse methods. For example, Winterton et al. (2010) analyzed the phylogeny and divergence time of Neuropterida through the morphological characteristics of each stage and four genes (*16S rDNA*, *18S rDNA*, *COI* and *CAD*) from 67 Neuropterida taxa [36]. Wang et al. (2017) illuminated the evolutionary history of Neuropterida by undertaking phylogenetic analyses of mitochondrial genomes of all families of Neuropterida [10]. Badano et al. (2017) performed the first particular quantitative phylogenetic analysis of Myrmeleontiformia using 107 larval morphological and behavioral characters from 36 genera [37].

The detailed information of the 10 clusters is summarized in Table 2. The silhouette index represents the quality. The closer the index to 1, the better was the cluster quality. The silhouette index above 0.7 represents good quality for all 10 clusters. The cluster map is shown in Figure 3. “Lacewing larva” was the biggest cluster (#0) composing 156 members. The most positive article was by Winterton et al. (2018) in this cluster, which reconstructed an advanced phylogenetic tree of Neuropterida using the anchored hybrid enrichment data. Many important conclusions were drawn in this study, such as Megaloptera being the sister group to Neuroptera and Coniopterygidae being the sister group to all other lacewings [12]. The second biggest cluster (#1) was “*Parallorhogas pyralophagus*”, which had 106 publications. The most positive article in this cluster was by Hilbeck et al. (1998), which studied the effects of transgenic maize on *B. thuringiensis* [38]. The third largest cluster (#2) was “transgenic insecticidal crop”, which had 92 publications. The most positive article in this cluster was by Romeis et al. (2004), which developed the assay to observe the effects of the Cry1Ab toxin [34].

#### 3.3.2. Author Co-Citation Analysis

**Figure 4:**
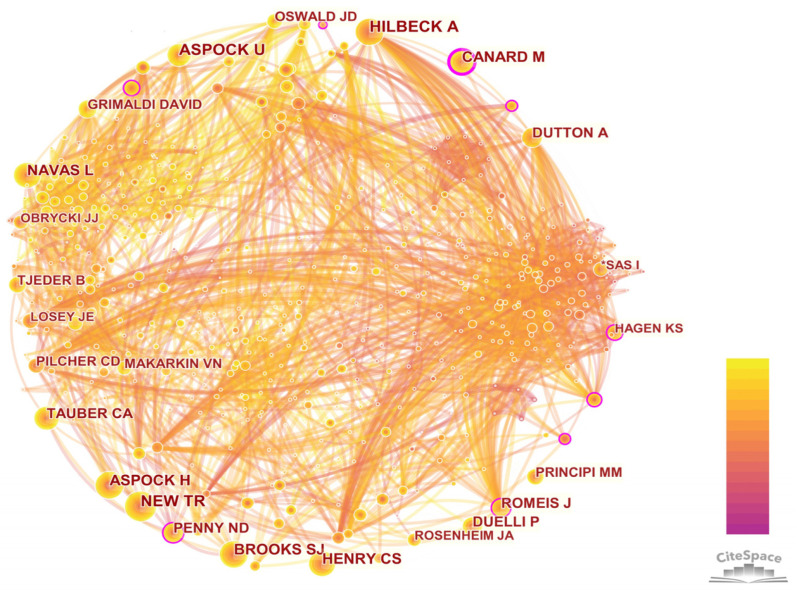
Author co-citation network. Each node stands for an author and each link depicts the interactions between authors. Node size represents the number of co-citations of the authors. Purple trims around some nodes indicate that those nodes received relatively high-centrality scores.

The network contained 548 authors and there were 3267 co-citation relationships (Figure 4). The top 10 authors in terms of frequency and centrality are presented in Table 3. The average citation frequency of individual authors was high. The top nine individuals were all cited more than 200 times, but the centrality was relatively low. The top five authors in terms of centrality were Yuyu Wang (0.23), Jörg Romeis (0.17), Michel Canard (0.15), Charles S. Henry (0.12) and Kenneth S. Hagen (0.1). It could be found that among them, the centrality was quite different. An analysis in terms of citation counts and centrality revealed that Michel Canard and Charles S. Henry were “core strength” researchers, whose research had important influences on this field. Michel Canard’s research fields were mainly about faunal analysis, biological control and behavior of Chrysopidae [41,42]. His most cited publication analyzed how lacewings adapt to seasonal changes [41]. Charles S. Henry’s research fields were mainly about species evolution and taxonomic identification of Chrysopidae, and whose most cited publications used song analysis, morphology and ecology characters to discover the true *C. carnea* [43].

#### 3.3.3. Journal Co-Citation Analysis

**Figure 5:**
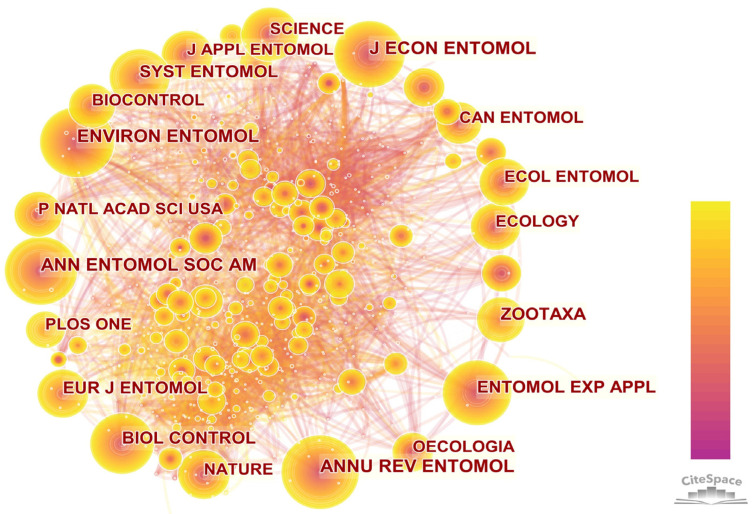
Journal co-citation network. The node size represents the co-citation frequency of each journal, and the connection line between nodes represents co-citation relationships.

The journal co-citation network contained 169 journals and 635 connections (Figure 5). The most cited journal was the *Annual Review of Entomology* (815 citations), but it had a low centrality (0.01). The reason may be that the journal is a review journal, which focuses on illustrating the progress and future trends in the research field of Neuropterida, but it does not put forward new research methods. The second most cited was the *Annals of the Entomological Society of America* (789 citations), but its centrality was also not high (0.02). It indicated that the co-citations with other journals were relatively low. From the basic analysis, *Biological Control* and the *European Journal of Entomology* were not only the journals with a higher number of publications, but also the most cited ones. In addition, they were the most widely published journals on Neuropterida. The impact factor of each journal in 2021 is illustrated in Table 4. Most of the top ten journals, in regard to the number of publications, had low impact factors which were not proportional to the number of publications. It showed that there are few high-level publications in the field, thus more studies are needed to focus on the highlights according to other insects. The impact factor of the *Annual Review of Entomology* was significantly higher than other journals and was the most frequently cited, which may be due to the journal having a representative position in the field of entomology and being recognized by many scholars.

### 3.4. The Collaboration Network Based on the Focus Topic

#### 3.4.1. Country Collaboration Analysis

**Figure 6:**
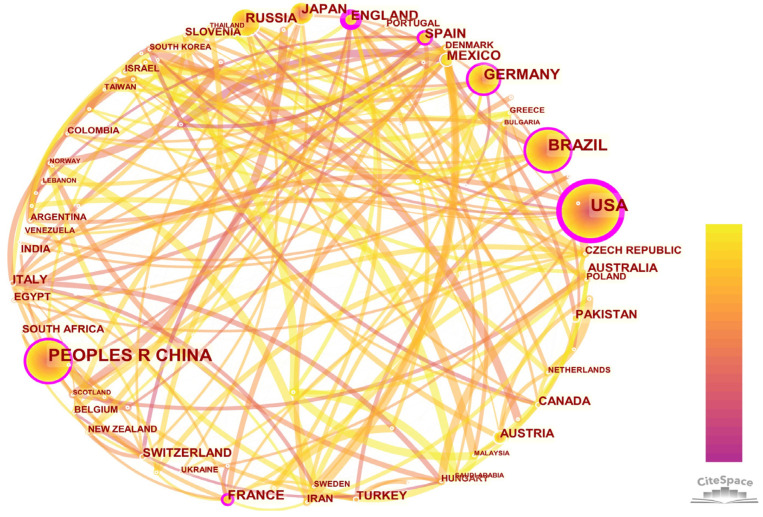
Country collaboration network. The node size represents the country’s publication frequency, and the connection line between nodes represents cooperation. High-centrality nodes are marked by a purple circle, which means the country has facilitated communication with other countries.

CiteSpace can realize the drawing of author, institution and international cooperation network graphics. By interpreting the graphics, it can be understood what the different levels of cooperation in the research field of Neuropterida are and discover the leading countries, institutions and individuals [31]. The map of cooperation between different countries contained 91 nodes and 381 connections (Figure 6). It showed that researchers from multiple countries attached importance to Neuropterida, and had multilateral cooperation among countries. Due to Neuropterida liking warm conditions, the countries that had more in-depth research on the Neuropterida were basically concentrated in tropical, subtropical and temperate regions. Among them, the United States not only started the earliest research on Neuropterida (1995), but also was much higher than other countries in the number of publications (675). In addition, the centrality was 0.52, which indicated that the United States had a leading position in the research field of Neuropterida. The research on the Neuropterida of China began in 2001. Although started relatively late, it developed rapidly. The number of publications was 437 and the centrality was 0.12, which indicated that there were a large number of publications from Chinese researchers, but the quality of publications needed to be improved. In addition, Brazil, Germany, Japan and Russia also had many excellent publications about Neuropterida. In recent years, the thickness of the annual ring had increased rapidly in many countries, which indicated that the number of publications had increased rapidly. The centrality of most European countries was high, which means, the same as with the United States, these countries had a large number of publications and collaborated with different nodes in the network. For example, England published 119 publications and its centrality was 0.31 (Table 5).

#### 3.4.2. Institution Collaboration Analysis

**Figure 7:**
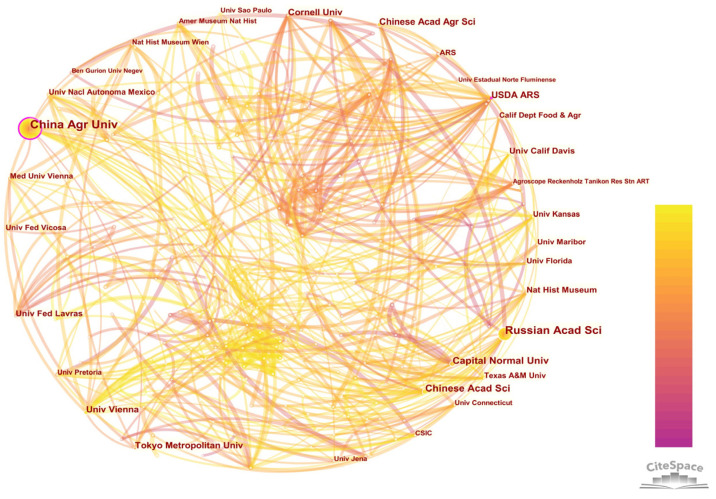
Institution collaboration network. The node size represents the institution’s publication frequency, and the connection line between nodes represents cooperation. High-centrality nodes are marked by a purple circle, which means the institution has facilitated communication with other institutions.

The institution collaboration network contained 537 institutions and 875 connections (Figure 7). Statistical data showed that most of the top 20 countries were agricultural countries (Table 6). The institution with the most publications was China Agricultural University and its first publication year was 2004. The earliest research of Neuropterida was the description of the *Coniocompsa Enderlein* (Coniopterygidae) in China [44]. China Agricultural University had a lot of cooperation with other institutions, whose research focused on the taxonomy and phylogeny of Neuropterida [10,45]. The early research on Neuropterida was mainly focused on the species identification and taxonomy [46,47]. With the development of science and technology, research of phylogenetic and evolution studies using modern techniques are more and more popular [10,48]. In addition, China Agricultural University has 201 publications, which are the most published publications in the field.

#### 3.4.3. Author Collaboration Analysis

The top 20 institutions with publications are mainly concentrated in the United States. It showed that the United States has a leading position in the research of Neuropterida. The United States Department of Agriculture-Agricultural Research Service centrality ranks the second in the world. It was the first institution (1995) to start this research. The earliest research of the United States Department of Agriculture-Agricultural Research Service on Neuropterida was about the improvement of the production system of lacewings [49]. The latest research was about the effects of biorational pesticide on aphids [50]. The Russian Academy of Sciences ranked second with only a fewer number of publications than China Agricultural University. The most frequently cited publication was a study on the remarkable Parakseneuridae and its phylogenetic position in primitive wing venation published in cooperation with Capital Normal University and other institutions in 2012 [41]. In the early stage of the Russian Academy of Sciences, it mainly studied the fossils and phylogenetic positions for the total purpose of Neuropterida by traditional methods [51].

**Figure 8:**
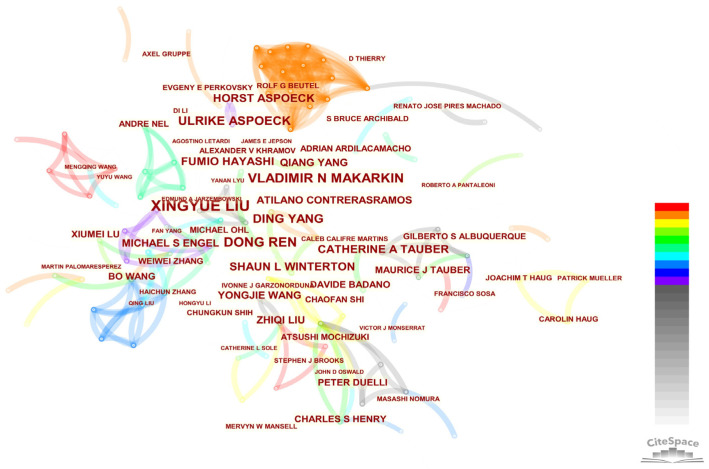
Author collaboration network. The node size represents the author’s publication frequency, and the connection line between nodes represents cooperation. Colors of the links, changing from light grey to red, display the corresponding formation year of the links between 1995 and 2021.

The author collaboration network in the research field of Neuropterida included 598 authors and 1032 connections (Figure 8). The early researchers were mostly individuals, and the cooperation was only between individuals. A representative was Catherine A. Tauber, whose main publication was “The genus *Ceraeochrysa* (Neuropterida: Chrysopidae) of America north of Mexico: Larvae, adults, and comparative biology” [52]. Modern research was often led by a small number of persons in charge, and a complex and close cooperation network was formed within the team. There was little cross-team communication, and the research teams were often composed by researchers from the same institution. Xingyue Liu, Vladimir N. Makarkin and Dong Ren were the top 3 authors that published the largest number of publications in the field of Neuropterida. Xingyue Liu ranked first in centrality (0.02) and published the most publications. Since 2004, Mario Waldburger had published only two publications on the Neuropterida, but his centrality was 0.01, indicating that his publications had important significance in recent years (Table 7).

### 3.5. The Emerging Trends of Neuropterida

#### 3.5.1. References Analysis with Citation Burst

**Figure 9:**
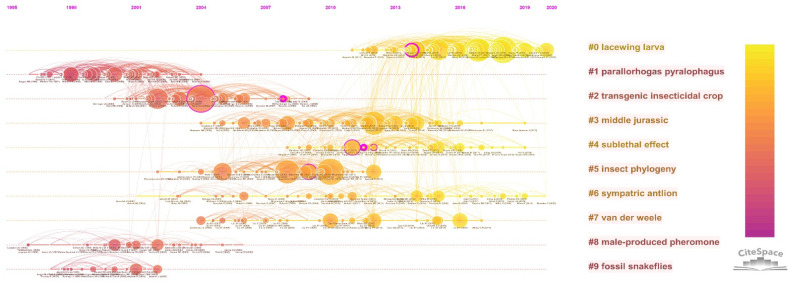
Timelines of co-citation clusters.

The literature co-citation network from the timeline perspective was obtained (Figure 9). It can be seen from the distribution of major clusters along the time axis that sympatric antlion and Middle Jurassic were long-term topics of Neuropterida research. The topic of lacewing larva appeared in recent years, and it continued to be a hot research topic until now. Parallorhogas pyralophagus, male-produced pheromone and fossil snakeflies were the earliest research topics. Then, studies of Neuropterida mainly focused on the behavioral, morphological and ecological significance prior to the extensive use of DNA sequencing technology. For example, Clark and Messina studied the effect of different plant structures on the predation behavior of lacewings in 1998 [53]. Hilbeck et al. studied the effects of Cry1Ab toxin through the indoor rearing of *C. carnea* [54]. Henry et al. explored the behavioral evolution of green lacewings by studying their courtship songs [55].

The reference burst was shown in Table 8. The publications widely cited in recent years were Shaun L. Winterton (2018) [12], Breitkreuz Laura C.V. (2017) [39], Michael S. Engel (2018) [33] and Yuyu Wang (2017) [10]. Among them, Shaun L. Winterton (2018) and Yuyu Wang (2017) had been introduced above. Breitkreuz et al. (2017) introduced the wing venation of Neuropterida, and some new ideas were put forward [39]. Engel et al. (2018) reviewed and summarized the phylogeny and evolution of Neuropterida over the past 25 years, putting forward four development prospects [33].

#### 3.5.2. Keyword Analysis with Citation Burst

**Figure 10:**
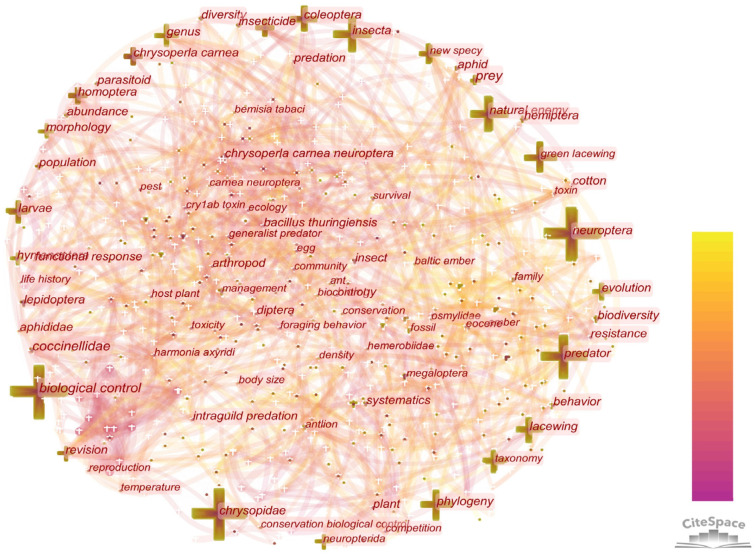
Co-occurring keywords map. The node size represents the keyword frequency. The line between nodes represents the co-occurrence between keywords.

The co-occurring keyword network was shown in Figure 10. The centrality measures the number of links between hot keywords and other keywords, which shows the power of the hot keywords in the network. High-frequency keywords can be used to identify hot topics in a research field and high-centrality keywords can reflect the status and influence of the hot topics. The top 10 keywords in terms of frequency and centrality are presented in Table 9. The hot keywords in the order of frequency and centrality were Neuroptera (frequency: 492, centrality: 0.08), biological control (frequency: 314, centrality: 0.08), Chrysopidae (frequency: 237, centrality: 0.07), and lacewing (frequency: 153, centrality: 0.06). Most of the high-frequency keywords appeared in the early 20th century. In recent years, there were fewer new high-frequency keywords.

The keyword bursts can be analyzed based on the citation frequency. “*Chrysoperla carnea*” and “Cry1Ab toxin” became keywords during the period of the literature bursts in this field and continued for a long time (Table 10). In recent years, “Neuroptera Chrysopidae”, “Evolution”, “Burmese amber”, “Myanmar”, “Mesozoic”, “Genera”, “Mitochondrial genome” and “Fossil” had become the bursting keywords. “Neuroptera Chrysopidae”, “Genera”, “Evolution” and “Mitochondrial genome” had emerged as keywords mainly because the Chrysopidae was a representative species of Neuropterida, which had significant importance in biocontrol and defining its phylogenetic position. “Burmese amber”, “Myanmar”, “Mesozoic” and “Fossil” became a focus, due to Neuropterida being one of the most primitive groups of Holometabola. The study of its fossil record was of great significance to Holometabola and even Insecta.

The latest hot topic related to the focus topic was the research on the phylogenetic position of Neuropterida based on the above publication and trend analysis. It mainly divided into two aspects of research, genomic data and fossil records. The phylogenetic studies of Neuropterida had come into a genomic era. With the continuous improvement of high-throughput sequencing technology and the gradual improvement of sequencing depth and accuracy in recent years, the research focus had gradually shifted to genome level research. The mitochondrial genome has many advantages in species analysis, such as simple operation and fast mutation rate [10,56,57,58,59,60,61,62], which were adequately used to resolve the phylogenetic and evolutionary history of Neuropterida. High-throughput transcriptome sequencing (RNA-seq) as well as next generation sequencing (NGS) has facilitated genome research [63,64,65,66]. The gene selection and evolutionary modeling would affect the phylogenomic inference of Neuropterida and using genes with strong phylogenetic signals may be an effective solution in phylogenetic reconstructions of Insecta [14]. The first chromosome-level genome of *C. pallens* as the first representative of Neuropterida, published by Yuyu Wang et al. (2021), revealed the potential molecular mechanisms as an excellent biocontrol agent and which provide an important genomic resource for future population genetics [23].

It was also a hot topic to study its phylogeny by observing the morphological characteristics of the fossil record. It was widely regarded as Holometabola since the early Permian of the Paleozoic Era, about 265 million years ago. Judging from the relatively small number of living species, the discontinuous distribution pattern and the rich fossil records, the most glorious period of evolution of the group has ended. Living groups were generally relict groups that have experienced a long geological history, and many of them could be considered as precious living fossils. For example, Liu et al. (2017) revisited the phylogenetic position of Corydasialidae based on a significant new fossil found in the Cretaceous amber of Myanmar [67]. Yang et al. (2012) analyzed the phylogenetic position of Parakseneuridae by using morphological data and DNA data for two ribosomal genes (*16S* and *18S rDNA*) and two protein-encoding genes (*COI* and *CAD*) from 33 families of Neuropterida [40].

## 4. Discussion

This research analyzed the relevant literature in the core database of WoS by using CiteSpace, and then summarized the beginning and development of the research in the field of Neuropterida. First, the number of publications published each year increased as well as decreased compared with the previous year, but it showed a trend of rapid growth in general from the time dimension. Since 2000, Neuropterida has become a research hotspot, and the number of publications has continued to grow steadily. The largest number of publications published was in 2019 with 172 publications. Second, the United States and China had the most publications on the Neuropterida and were the main countries for research. These two countries have had the most productive authors and institutions. There was a great deal of academic cooperation between them. The leading position of the United States and China in this field may be due to the fact that both countries are large agricultural countries, and Neuropterida is the natural enemy of many pests. Since 2000, China’s economy has developed rapidly and demand for land has continued to increase. Year-round continuous cropping has made the soil prone to outbreaks of diseases and insect pests, so related research has developed rapidly. Representative universities for research on natural enemies of Neuropterida included the China Agricultural University and the Chinese Academy of Agricultural Sciences. Due to the late start of Neuropterida research in China, the early research focused on practical applications. For example, Qi et al. (2001) showed that neem-fed predatory insects affect Neuropterida and Coleoptera, but the phylogenetic relationship between species has not been investigated in depth [68]. Finally, most of the research belongs to the field of entomology and ecology, based on the research content. In addition, there is more cross-research in the fields of agriculture and biochemistry, molecular biology, chemistry, etc., indicating that the research of Neuropterida can be covered from the macroscopic study to the microscopic study.

As the citation burst analysis shows, most of the high-frequency keywords appeared in the early 20th century, whereas there were fewer new high frequency keywords in recent years. Combined with the more detailed publication interpretation in keywords analysis, it showed that the research progress in the field of Neuropterida was closely related to the level of scientific and technological development. With the continuous improvement of high-throughput sequencing technology in recent years and the gradual improvement of sequencing depth and accuracy, research hotspots have gradually shifted to molecular research, including the study of the phylogenetic evolution, and the classification of Neuropterida using molecular techniques. It can be predicted that the future research of Neuropterida will focus on the genomic studies and molecular mechanisms of their morphological characters, behavior, historical evolution and so on, as the first chromosome-level genome of *C. pallens* has been published. With the development of molecular technology, the interspecies relationships between Neuropterida and other insects such as their prey will also become research hotspots.

Despite the rapid development of molecular research, many other areas, including the distribution of species in the spatial structure of ecosystems and interactions between species (competition), also attracted a lot of attention. For example, Megaloptera are aquatic insects in terms of larval physiology and behavior, but not in the ecological sense, as they have not been fully studied in many temperate and tropical benthic communities [69]. People can formulate reasonable aphid control strategies by observing the trend of seasonal population changes of green lacewings on sorghum, as green lacewings are the natural enemies of aphids [70]. Due to the crush of rocks, mantidflies (Neuroptera: Mantispidae) fossils are so rare that the evolution of their raptorial legs is still largely assumed or inferred by phylogeny [71]. At present, there are still many weak aspects of the research on Neuropterida. For example, research on the internal anatomy of Neuropterida is very scarce, which is easy to be ignored. In addition, the study of Neuropterida fossils also needs more comprehensive analyses, which will benefit the study of the biology, ecology, phylogeny and evolution of the extant species. Raphidioptera has abundant morphological diversity, but few studies have been conducted. With the increase of Raphidioptera fossils, the study of Raphidioptera will also become a hot topic [33,72].

## 5. Conclusions

In conclusion, Neuropterida has become a research hotspot, and the number of publications has continued to grow steadily since 2000. The United States and China had the most publications on the Neuropterida and were the main countries for research. Representative institutions for research on natural enemies of Neuropterida included the China Agricultural University and the Chinese Academy of Agricultural Sciences. Most of the research belongs to the fields of entomology and ecology, in regard to the research content. Xingyue Liu, Vladimir N. Makarkin and Dong Ren were the top three authors who published the largest number of publications in the field of Neuropterida. With the continuous improvement of high-throughput sequencing technology in recent years and the gradual improvement of sequencing depth and accuracy, research hotspots have gradually shifted to molecular research, including the study of the molecular mechanisms of their morphological characters, behavior, historical evolution and so on. In addition, the seasonal dynamics of species and populations, rarity of species and competition between species are also a research focus of Neuropterida.

## Figures and Tables

**Table 1:** Top 10 most cited articles about Neuropterida.

Citation Counts	Title	Author	Year	Centrality	Journal	Cluster #
61	Evolution of lacewings and allied orders using anchored phylogenomics (Neuroptera, Megaloptera, Raphidioptera) [12]	Shaun L. Winterton	2018	0.02	*Systematic Entomology*	0
50	Phylogenetic relevance of the genital sclerites of Neuropterida (Insecta: Holometabola) [7]	Ulrike Aspöck	2008	0.02	*Systematic Entomology*	5
50	Wing tracheation in Chrysopidae and other Neuropterida (Insecta): a resolution of the confusion about vein fusion [39]	Laura C. V. Breitkreuz	2017	0.02	*American Museum Novitates*	0
48	Phylogeny and evolution of Neuropterida: where have wings of lace taken us? [33]	Michael S. Engel	2018	0.01	*Annual Review of Entomology*	0
47	Mitochondrial phylogenomics illuminates the evolutionary history of Neuropterida [10]	Yuyu Wang	2017	0.02	*Cladistics*	0
45	Bacillus thuringiensis toxin (Cry1Ab) has no direct effect on larvae of the green lacewing *Chrysoperla carnea* (Stephens) (Neuroptera: Chrysopidae) [34]	Jörg Romeis	2004	0.19	*Journal of Insect Physiology*	2
45	On wings of lace: phylogeny and Bayesian divergence time estimates of Neuropterida (Insecta) based on morphological and molecular data [36]	Shaun L. Winterton	2010	0.03	*Systematic Entomology*	5
43	A remarkable new family of Jurassic Insects (Neuroptera) with primitive wing venation and its phylogenetic position in Neuropterida [40]	Qiang Yang	2012	0.03	*PLoS ONE*	3
37	Uptake of Bt-toxin by herbivores feeding on transgenic maize and consequences for the predator *Chrysoperla carnea* [35]	Anna Cristina Dutton	2002	0.10	*Ecological Entomology*	2
35	Phylogeny of Myrmeleontiformia based on larval morphology (Neuropterida: Neuroptera) [37]	Davide Badano	2017	0.01	*Systematic Entomology*	0

**Table 2:** Top-ranked clusters about Neuropterida.

Cluster ID	Size	Silhouette	Label (LLR)	Mean Year
0	156	0.902	Lacewing larva	2016
1	106	0.951	Parallorhogas pyralophagus	2000
2	92	0.935	Transgenic insecticidal crop	2004
3	91	0.874	Middle Jurassic	2010
4	84	0.902	Sublethal effect	2013
5	71	0.891	Insect phylogeny	2007
6	62	0.977	Sympatric antlion	2011
7	42	0.96	Van der Weele	2009
8	39	0.98	Male-produced pheromone	2001
9	31	1	Fossil snakeflies	1999

**Table 3:** Top 10 authors in terms of frequency and centrality.

Author	Frequency	Centrality	Author	Centrality	Frequency
Ulrike Aspöck	348	0.04	Yuyu Wang	0.23	91
Horst Aspöck	280	0.08	Jörg Romeis	0.17	87
T. R. New	271	0.03	Michel Canard	0.15	180
L. Navás	268	0.01	Charles S. Henry	0.12	178
John D. Oswald	243	0.03	K. S. Hagen	0.10	57
Vladimir N. Makarkin	226	0.04	Horst Aspöck	0.08	280
Shaun L. Winterton	220	0.02	Stephen J. Brooks	0.08	208
Xingyue Liu	216	0.03	Maurice J. Tauber	0.08	99
Stephen J. Brooks	208	0.08	Gilberto S. Albuquerque	0.08	74
Catherine A. Tauber	195	0.06	T. Eisner	0.08	56

**Table 4:** Top 10 journals based on cited frequency.

Journals	Frequency	Centrality	Impact Factor
*Annual Review of Entomology*	815	0.01	19.686
*Annals of the Entomological Society of America*	789	0.02	2.099
*Environmental Entomology*	747	0.01	2.377
*Journal of Economic Entomology*	669	0.01	2.381
*Biological Control*	663	0.03	3.687
*Systematic Entomology*	631	0.02	3.844
*Entomologia Experimentalis et Applicata*	561	0.01	2.25
*Zootaxa*	534	0.01	1.091
*European Journal of Entomology*	491	0.04	1.225
*PLoS ONE*	460	0.01	3.24

**Table 5:** Top 10 countries based on cited frequency.

Rank	Frequency	Country	Centrality	Country
1	642	United States	0.32	USA
2	471	China	0.31	England
3	287	Brazil	0.19	France
4	176	Germany	0.16	Spain
5	138	Russia	0.15	Germany
6	129	Japan	0.11	China
7	119	England	0.10	Brazil
8	102	Spain	0.09	Switzerland
9	101	Mexico	0.09	Italy
10	99	France	0.07	Australia

**Table 6:** Top 20 institutions based on occurred frequency.

Institutions	Frequency	Centrality
China Agricultural University	224	0.15
Russian Academy of Sciences	129	0.06
Capital Normal University	85	0.04
Chinese Academy of Sciences	76	0.05
Cornell University	62	0.08
Chinese Academy of Sciences	57	0.04
University of Vienna	53	0.04
United States Department of Agriculture-Agricultural Research Service	49	0.12
Tokyo Metropolitan University	47	0.01
Natural History Museum	45	0.09
University of California Davis	43	0.04
Universidade Federal de Lavras	38	0.04
Medical University of Vienna	31	0.01
University of Kansas	30	0.02
California Department of Food and Agriculture	30	0.01
Universidad Nacional Autonoma de Mexico	30	0.03
Florida State University	28	0.04
University of Maribor	27	0.01
Universidade Federal de Vicosa	25	0.03
Texas A&M University	24	0.02

**Table 7:** Top 10 authors in terms of frequency and centrality.

Author	Frequency	Centrality	Author	Centrality	Frequency
Xingyue Liu	165	0.02	Xingyue Liu	0.02	165
Vladimir N. Makarkin	75	0.01	Peter Duelli	0.02	17
Dong Ren	67	0	Jörg Romeis	0.02	16
Ulrike Aspöck	57	0.01	Mario Waldburger	0.02	2
Ding Yang	55	0	Vladimir N. Makarkin	0.01	75
Horst Aspöck	45	0	Ulrike Aspöck	0.01	57
Catherine A. Tauber	40	0.01	Catherine A. Tauber	0.01	40
Fumio Hayashi	40	0	Shaun L. Winterton	0.01	37
Shaun L. Winterton	37	0.01	Atilano Contrerasramos	0.01	27
Yongjie Wang	31	0	Michael S. Engel	0.01	26

**Table 8:** Top 10 references based on citation burst strength.

References	Year	Strength	Begin	End	1995–2021
Shaun L. Winterton, 2018, *Systematic Entomology*, V43, P330 [12]	2018	26.13	2018	2021	▂▂▂▂▂▂▂▂▂▂▂▂▂▂▂▂▂▂▂▂▂▂▂ ▃▃▃▃
Aspöck Ulrike, 2008, *Systematic Entomology*, V33, P97 [7]	2008	25.47	2009	2013	▂▂▂▂▂▂▂▂▂▂▂▂▂▂ ▃▃▃▃▃ ▂▂▂▂▂▂▂▂
Jörg Romeis, 2004, *Journal of Insect Physiology*, V50, P175 [34]	2004	21.72	2005	2009	▂▂▂▂▂▂▂▂▂▂ ▃▃▃▃▃ ▂▂▂▂▂▂▂▂▂▂▂▂
Shaun L. Winterton, 2010, *Systematic Entomology*, V35, P349 [36]	2010	21.61	2011	2015	▂▂▂▂▂▂▂▂▂▂▂▂▂▂▂▂ ▃▃▃▃▃ ▂▂▂▂▂▂
Laura C.V. Breitkreuz, 2017, *American Museum Novitates*, V3890, P1 [39]	2017	21.36	2018	2021	▂▂▂▂▂▂▂▂▂▂▂▂▂▂▂▂▂▂▂▂▂▂▂ ▃▃▃▃
Michael S. Engel, 2018, *Annual Review of Entomology*, V63, P531 [33]	2018	20.5	2018	2021	▂▂▂▂▂▂▂▂▂▂▂▂▂▂▂▂▂▂▂▂▂▂▂ ▃▃▃▃
Yuyu Wang, 2017, *Cladistics*, V33, P617 [10]	2017	20.06	2018	2021	▂▂▂▂▂▂▂▂▂▂▂▂▂▂▂▂▂▂▂▂▂▂▂ ▃▃▃▃
Qiang Yang, 2012, *PLoS ONE*, V7 [40]	2012	18.64	2013	2017	▂▂▂▂▂▂▂▂▂▂▂▂▂▂▂▂▂▂ ▃▃▃▃▃ ▂▂▂▂
Anna Dutton, 2002, *Ecological Entomology*, V27, P441 [35]	2002	18.23	2003	2007	▂▂▂▂▂▂▂▂ ▃▃▃▃▃ ▂▂▂▂▂▂▂▂▂▂▂▂▂▂
Angelika Hilbec, 1998, *Environmental Entomology* [30]	1998	17.16	2001	2003	▂▂▂▂▂▂ ▃▃▃ ▂▂▂▂▂▂▂▂▂▂▂▂▂▂▂▂▂▂

Note: There are 27 short lines, representing each year from 1995 to 2021. The red part represents the year in which the citation frequency of the reference burst from the beginning to the end.

**Table 9:** Top 10 keywords in terms of frequency and centrality.

Rank	Frequency	Keywords	Centrality	Keywords
1	492	Neuroptera	0.08	Neuroptera
2	314	Biological control	0.08	Evolution
3	237	Chrysopidae	0.08	Prey
4	233	Insecta	0.08	Hymenoptera
5	229	Predator	0.07	Biological control
6	204	Phylogeny	0.07	Chrysopidae
7	200	Natural enemy	0.07	Coleoptera
8	172	Green lacewing	0.07	Diptera
9	153	Lacewing	0.06	Lacewing
10	150	Coleoptera	0.06	*Chrysoperla carnea*

**Table 10:** Top 20 keywords based on citation burst strength.

Keywords	Strength	Begin	End	1995–2021
*Chrysoperla carnea*	13.64	2001	2007	▂▂▂▂▂▂ ▃▃▃▃▃▃▃ ▂▂▂▂▂▂▂▂▂▂▂▂▂▂
Cry1ab toxin	11.28	2001	2008	▂▂▂▂▂▂ ▃▃▃▃▃▃▃▃ ▂▂▂▂▂▂▂▂▂▂▂▂▂
Neuroptera Chrysopidae	10.1	2017	2021	▂▂▂▂▂▂▂▂▂▂▂▂▂▂▂▂▂▂▂▂▂▂ ▃▃▃▃▃
*Bacillus thuringiensis*	9.52	2001	2007	▂▂▂▂▂▂ ▃▃▃▃▃▃▃ ▂▂▂▂▂▂▂▂▂▂▂▂▂▂
*Bacillus thuringiensis* corn	9.28	2001	2008	▂▂▂▂▂▂ ▃▃▃▃▃▃▃▃ ▂▂▂▂▂▂▂▂▂▂▂▂▂
Transgenic plant	8.45	2001	2006	▂▂▂▂▂▂ ▃▃▃▃▃▃ ▂▂▂▂▂▂▂▂▂▂▂▂▂▂▂
Family	8.29	2009	2015	▂▂▂▂▂▂▂▂▂▂▂▂▂▂ ▃▃▃▃▃▃▃ ▂▂▂▂▂▂
Evolution	8.23	2018	2021	▂▂▂▂▂▂▂▂▂▂▂▂▂▂▂▂▂▂▂▂▂▂▂ ▃▃▃▃
Burmese amber	7.56	2017	2021	▂▂▂▂▂▂▂▂▂▂▂▂▂▂▂▂▂▂▂▂▂▂ ▃▃▃▃▃
Myanmar	7.4	2015	2021	▂▂▂▂▂▂▂▂▂▂▂▂▂▂▂▂▂▂▂▂ ▃▃▃▃▃▃▃
Mesozoic	7.33	2016	2021	▂▂▂▂▂▂▂▂▂▂▂▂▂▂▂▂▂▂▂▂▂ ▃▃▃▃▃▃
*Coleomegilla maculata* Coleoptera	6.85	2005	2010	▂▂▂▂▂▂▂▂▂▂ ▃▃▃▃▃▃ ▂▂▂▂▂▂▂▂▂▂▂
Genera	6.85	2018	2021	▂▂▂▂▂▂▂▂▂▂▂▂▂▂▂▂▂▂▂▂▂▂▂ ▃▃▃▃
Host plant	6.35	2011	2016	▂▂▂▂▂▂▂▂▂▂▂▂▂▂▂▂ ▃▃▃▃▃▃ ▂▂▂▂▂
Mitochondrial genome	6.21	2015	2021	▂▂▂▂▂▂▂▂▂▂▂▂▂▂▂▂▂▂▂▂ ▃▃▃▃▃▃▃
Fossil	5.86	2018	2021	▂▂▂▂▂▂▂▂▂▂▂▂▂▂▂▂▂▂▂▂▂▂▂ ▃▃▃▃
Noctuidae	5.79	2002	2006	▂▂▂▂▂▂▂ ▃▃▃▃▃ ▂▂▂▂▂▂▂▂▂▂▂▂▂▂▂
Aphididae	5.73	2010	2013	▂▂▂▂▂▂▂▂▂▂▂▂▂▂▂ ▃▃▃▃ ▂▂▂▂▂▂▂▂
Corydalidae	5.72	2004	2008	▂▂▂▂▂▂▂▂▂ ▃▃▃▃▃ ▂▂▂▂▂▂▂▂▂▂▂▂▂
Ecology	5.71	2001	2007	▂▂▂▂▂▂ ▃▃▃▃▃▃▃ ▂▂▂▂▂▂▂▂▂▂▂▂▂▂

Note: There are 27 short lines, representing each year from 1995 to 2021. The red part represents the year in which the citation frequency of the keyword burst from the beginning to the end.

## Data Availability

All data generated or analyzed during this study are included in this published article.
